# Conditional Gene Expression in *Chlamydia trachomatis* Using the Tet System

**DOI:** 10.1371/journal.pone.0076743

**Published:** 2013-10-07

**Authors:** Jason Wickstrum, Lindsay R. Sammons, Keasha N. Restivo, P. Scott Hefty

**Affiliations:** Department of Molecular Biosciences, University of Kansas, Lawrence, Kansas, United States of America; Oregon State University, United States of America

## Abstract

*Chlamydia trachomatis* is maintained through a complex bi-phasic developmental cycle that incorporates numerous processes that are poorly understood. This is reflective of the previous paucity of genetic tools available. The recent advent of a method for transforming *Chlamydia* has enabled the development of essential molecular tools to better study these medically important bacteria. Critical for the study of *Chlamydia* biology and pathogenesis, is a system for tightly controlled inducible gene expression. To accomplish this, a new shuttle vector was generated with gene expression controlled by the Tetracycline repressor and anhydryotetracycline. Evaluation of GFP expression by this system demonstrated tightly controlled gene regulation with rapid protein expression upon induction and restoration of transcription repression following inducer removal. Additionally, induction of expression could be detected relatively early during the developmental cycle and concomitant with conversion into the metabolically active form of *Chlamydia*. Uniform and strong GFP induction was observed during middle stages of the developmental cycle. Interestingly, variable induced GFP expression by individual organisms within shared inclusions during later stages of development suggesting metabolic diversity is affecting induction and/or expression. These observations support the strong potential of this molecular tool to enable numerous experimental analyses for a better understanding of the biology and pathogenesis of *Chlamydia*.

## Introduction

*Chlamydia trachomatis* infections have an immense impact on public health as the most common sexually transmitted bacteria and most prevalent cause of preventable blindness worldwide. These obligate intracellular bacteria are maintained through a characteristic bi-phasic developmental cycle that is strongly tethered to pathogenesis (reviewed in 1,2). The extracellular infectious form, elementary body (EB), predominantly infects epithelial cells of the genital tract. After facilitating entry into a host cell, they quickly establish the *Chlamydia* specific parasitophorous vacuole referred to as an inclusion. Within the inclusion, EBs undergo conversion to a metabolically active and replicative form known as a reticulate body (RB). The inclusion grows in size as replication continues and starting around 18-24 hours after entry into the host cell, portions of RBs asynchronously convert into EBs. This process continues until release from the host cell, through lysis or extrusion [3], and infectious EBs establish a new infection. Throughout this developmental cycle, the organism manipulates the host cell in order to maintain its intercellular environment, acquire nutrients, and coordinate replication, conversion, and exit processes. Importantly, the components and processes to accomplish these diverse biological goals are still poorly understood.

The slow progress in understanding many components associated with the basic biology and pathogenesis is reflective of long-standing limitations in the *Chlamydia* field; the inability to introduce stably maintained DNA and perform targeted gene manipulation. One of these limitations was removed by the development of a method for introducing DNA and the modification of the native plasmid to allow effective selection of transformants [4]. This accomplishment has enabled the development of key molecular tools for studying *Chlamydia*. Recently, Agaisse and Derre [5] utilized this transformation system to test the expression of a diverse set of fluorescent proteins and demonstrate utility for sub-cellular localization studies. However, a tool that has yet to be developed in *Chlamydia* is one allowing the ability to carefully control gene expression. This molecular tool is critical for analyzing the biologic effect of a given gene product and is especially applicable for *Chlamydia* as precise gene regulation at specific points in the developmental cycle will be key to delineate biological role and function.

There are numerous methods for artificially controlling gene expression, each with many strengths, limitations, and applications ( [6]; for review). One widely used system for carefully controlled gene expression relies on the tetracycline repressor (TetR) [7,8]. TetR binds tightly to *tet* operators (*tetO*_*1*_ and *tetO*_*2*_) within the *tetA* promoter strongly inhibiting transcription. Typically, as tetracycline is introduced, it binds to TetR and relieves repression of transcription. Expression systems controlled by TetR have been utilized in many gram-negative and gram-positive bacteria [9-12] as well as to control gene expression of bacteria in eukaryotic cells and in mice [13-15]. This is largely due to the ability of tetracyclines to cross diverse biological membranes effectively. Furthermore, TetR regulated systems have displayed a wide dynamic range (~5000 fold) following induction [11]. Importantly, the system can be induced with anhydrotetracycline (ATc), a chemical analog of tetracycline but with higher affinity (~30 fold) for TetR requiring much lower concentration for de-repression [16]. Additionally, ATc is an atypical tetracycline with different and limited antibacterial effects relative to tetracycline [17]. This is a key aspect as *Chlamydia* is very sensitive to tetracycline. Lastly, the Tet system has the future potential flexibility for the use of the revTetR repressor that allows for inducible repression at sites that contain the *tetO* sequences [18].

This study was designed to leverage the previous observations that the native chlamydial plasmid can be effectively engineered as a shuttle vector. The native L2 plasmid was modified by introducing another plasmid (pASK-IBA33plus) containing Tet inducible gene expression components. Green fluorescent protein (GFP) was utilized to monitor and measure the response and activity of the inducible system to determine many of the basic parameters for induction. Additionally, a red fluorescent protein (mKate2) under the control of a constitutive promoter was added to the shuttle vector to facilitate normalization and enable fluorescence comparisons within individual cells.

## Results

### Development of Tet controlled shuttle vector for *Chlamydia trachomatis*

**Figure 1:**
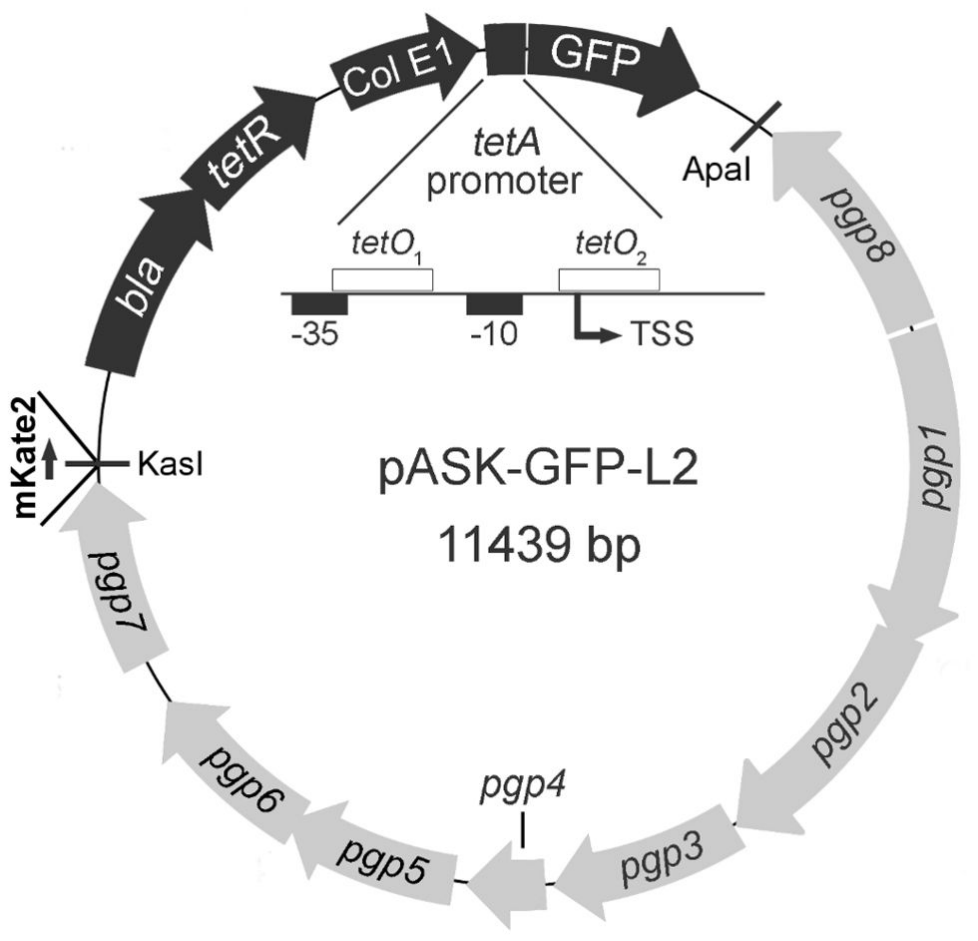
Shuttle vector with GFP expression under Tet control. The *E*. *coli* cloning vector pASK-IBA33plus, with GFP inserted under Tet control, was ligated to the wild-type plasmid from *C*. *trachomatis* strain L2 using PCR-generated restriction endonuclease sites (see Materials and Methods for details). A variation of this shuttle vector was later constructed with a gene encoding mKate2 inserted at the indicated *Kas*I site.

Multiple *C. trachomatis* shuttle vectors have been developed and stably maintained by *C. trachomatis* [4,5,19-21]. Many of these have utilized a conserved unique *Bam*HI site for convenient cloning; however, genetic introduction at this site disrupts *pgp7*. While this disruption appears to have limited effect on *in vitro* (tissue culture) [19] and *in vivo* (mouse) infections, the region between the converging *pgp7* and *pgp8* genes was targeted for cloning to minimize unanticipated complications. The commercially available pASK-IBA33plus parent vector is pASK75 in which *tetR* was transcriptionally fused to the constitutively expressed β-lactamase encoding gene [22]. pGFP::SW2 [4] was used as template for cloning GFP into the MCS to generate pASK-GFP. This GFP is a S65T variant of the original GFP [23] and previously demonstrated to be functional in *Chlamydia* [4]. This vector was cloned into the native *C. trachomatis* L2 plasmid creating the shuttle vector pASK-GFP-L2 (Figure 1). GFP expression from pASK-GFP-L2 in response to addition of ATc in *E. coli* was observed by microscopy (data not shown).

### *C. trachomatis* sensitivity to ATc

**Figure 2:**
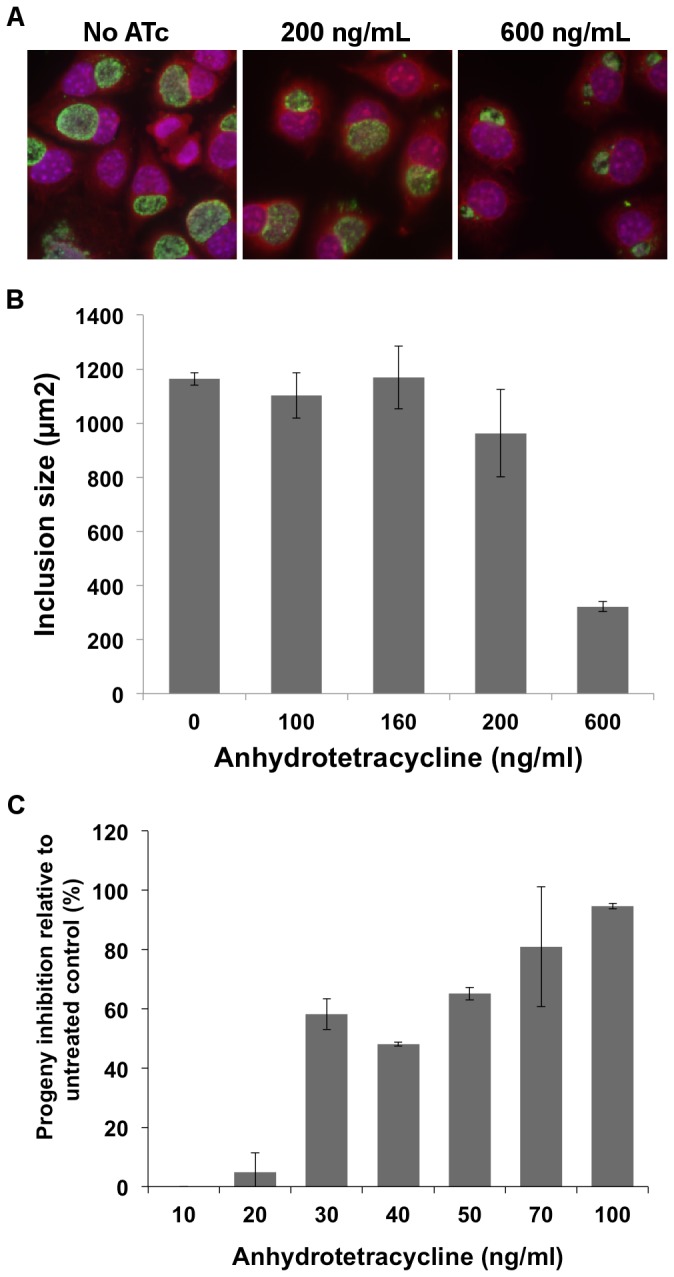
Effect of anhydrotetracycline on *C. trachomatis*. L929 cells were infected with wild-type *C*. *trachomatis* L2 and then incubated in the presence of various concentrations of ATc throughout the infection (0 to 24 hpi). Cultures were fixed at 24 hpi and stained for MOMP (green). Representative images from three different concentrations are shown (A). Additionally, the same samples (and additional samples) were analyzed using Cell Profiler to calculate the average inclusion size (B). Over 700 inclusions were analyzed at each concentration. Separately, infected L929 cell cultures were treated with different concentrations of ATc and lysed at 24 hpi. Lysates were used to infect a fresh monolayer (in the absence of ATc) and infection levels were enumerated at 24 hpi using immunofluorescence microscopy. Percent inhibition of progeny was determined relative to a lysate from an untreated control (C).

Prior to introducing pASK-GFP-L2 into *C. trachomatis*, sensitivity to the antibiotic ATc was evaluated given the relatively high sensitivity of Chlamydia to tetracycline [24,25]. In other diverse bacteria (*Mycobacterium*, *Borrelia*, *E. coli*, and *Staphyloccus*), final concentrations of ATc ranging from 0.25 to 20.0 µg/ml induced gene expression without apparent negative effects [9-12]. To gain a better appreciation of the sensitivity of *Chlamydia* to ATc, a wide range of concentrations was incubated with *C. trachomatis* for 24 hours and inclusions were evaluated. At 200 ng/ml or less, little to no effect on the number or size of inclusions was detected. However, at 600 ng/mL, infection and growth of *Chlamydia* was severely affected (Figure 2A and 2B). The ability of *C. trachomatis* to produce infectious progeny after 24 hours of exposure to ATc was also assessed. Surprisingly, close to a 50% reduction in infectious progeny was detected at ATc concentrations as low as 30 ng/mL (Figure 2C). Importantly, no or slight effect on progeny production was detected at 10 to 20 ng/ml, respectively.

### The Tet control system is functional in *C. trachomatis*

**Figure 3:**
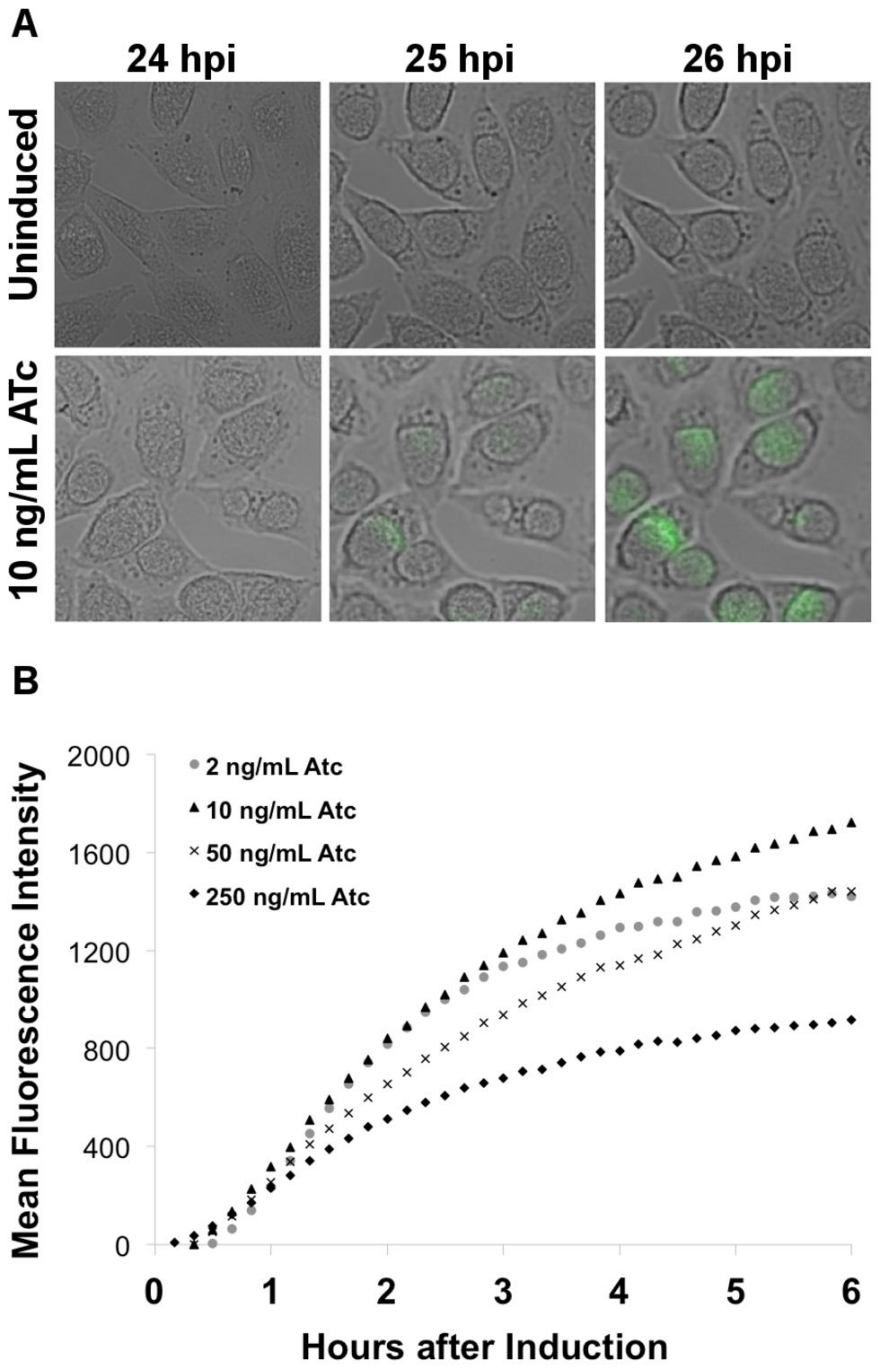
GFP expression after induction at 24 hpi. L929 cell cultures infected with pASK-GFP-L2 transformed *C*. *trachomatis* were induced with various concentrations of ATc at 24 hpi. Images were captured every 10 minutes for a total of 6 hours. Representative images from the culture induced with 10 ng/mL and an uninduced control are shown (A). The images were also analyzed using the Otsu segmentation algorithm to determine the mean fluorescence intensities in arbitrary units within the inclusions (B). Three microscope fields were averaged for each sample with approximately 40 infected cells per field. The mean fluorescence intensity of an uninduced sample was also calculated at each time point and the resulting values were subtracted from the induced samples at the corresponding time points.

*C. trachomatis* was transformed with the pASK-GFP-L2 shuttle vector and transformants were selected. Live imaging with fluorescent microscopy was used to monitor GFP expression in L929 cells infected with transformed *C. trachomatis*. At 24 hours post infection, when normal inclusions were readily detectable by microscopy, final concentrations of ATc ranging from 2 to 250 ng/mL were added to the cultures and live images taken for 6 hours after induction. In the absence of ATc, GFP expression was not detectable by manual visualization; however, there was evident GFP expression within 1 hour of induction with 10 ng/mL ATc and a marked increase after 2 hours (Figure 3A and Video S1). GFP fluorescence intensity from microscope images was quantified over the entire 6 hours of ATc induction. The resulting analysis showed similar levels of GFP expression throughout the 6 hours by induction with 2, 10, and 50 ng/mL ATc (Figure 3B). While the expression rates and levels were similar between 2-50 ng/mL, the rate of GFP expression was clearly slower when the concentration was adjusted to 50 ng/mL, indicating possible toxic ATc effects. Even more pronounced, by 2 hours post-induction, the 250 ng/mL samples began to exhibit much lower fluorescence, relative to the other induced samples, reflecting a negative effect on *Chlamydia* cell function. These initial observations demonstrate that ATc was able to diffuse across four diverse membranes; the host cell membrane, the inclusion membrane, and the *Chlamydia* outer and inner membrane to effectively relieve repression by the Tet repressor. These results also support that the Tet repressor gene was being expressed from the shuttle vector (*bla* promoter) and the *tetA* promoter was functional and responding as anticipated. Lastly, these data, in combination with the results in Figure 2, also support that ATc concentrations at 30 ng/mL may be near the upper limit for induction without apparent negative effects for expression studies

### Bacterial cell to cell variation following induction

**Figure 4:**
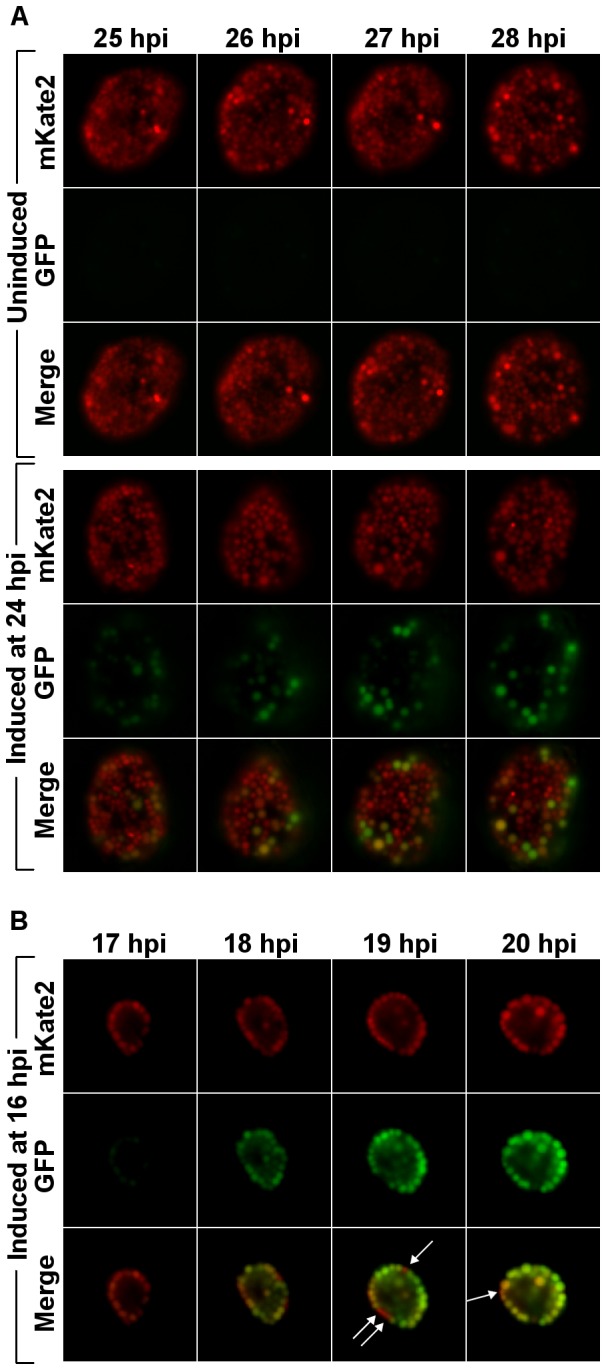
Detection of constitutive mKate2 and inducible GFP. L929 cell cultures infected with pASK-GFP/mKate2-L2 transformed *C*. *trachomatis* were induced at 24 hpi and images were then taken at indicated time points. Representative images from an uninduced sample and a sample induced with 10 ng/mL ATc at 24 hpi are shown in (A). Representative images of a sample induced with 10 ng/mL ATc at 16 hpi (from a separate experiment) are shown in (B). White arrows highlight the relatively few *C*. *trachomatis* cells within the inclusion that did not express detectable GFP at 19 hpi and 20 hpi.

In the fluorescent microscopy images shown in Figure 2A and other results using higher magnification (Video S1), it appeared that individual *C. trachomatis* cells within an inclusion were not being uniformly induced. It was not clear if some cells were losing the shuttle vector, if the inducer was not evenly distributed inside the inclusion, or if some cells were not in a proper metabolic state to take up the inducer and express GFP. To help address these possibilities, a second shuttle vector, pASK-GFP/mKate2-L2 (Figure 1), was constructed and used to transform *C. trachomatis*. Similar to pASK-GFP-L2, this new shuttle vector placed GFP expression under Tet control, but it also contained a gene encoding the red fluorescent protein mKate2 [26] under control of a mutated core *dnaK* promoter (see Materials and Methods for details) as a marker of metabolically active, transformed *C. trachomatis*. *C. trachomatis* transformed with pASK-GFP/mKate2-L2 was used to infect L929 cells and ATc at a final concentration of 10 ng/mL was added at 24 hpi. The images in Figure 4A (see also Video S2) show mKate2 expression in *C. trachomatis* cells throughout the inclusion, but highly variable GFP expression after addition of inducer. The mostly uniform mKate2 expression suggests that the *C. trachomatis* cells had the shuttle vector, but many were not expressing GFP after addition of inducer. Induction at 24 hpi with lower or higher concentrations of ATc was tested and the variation in GFP expression within inclusions was still observed (not shown).

The variable expression was hypothesized to primarily reflect the diversity of metabolic states of *Chlamydia* at 24 hpi. Therefore it was expected that induction at earlier time points, when most, if not all, organisms are still in the RB replicative stage of development, would result in more uniform induction. *C. trachomatis* transformants containing the pASK-GFP/mKate2-L2 shuttle vector were induced with 10 ng/mL ATc starting at 16 hpi and images were taken at various time points during incubation (Figure 4B; see also Video S3). With this earlier induction period, GFP expression was substantially more uniform within inclusions and matched mKate2 expression much more closely than induction at 24 hpi.

### Quantification of total GFP induction

**Figure 5:**
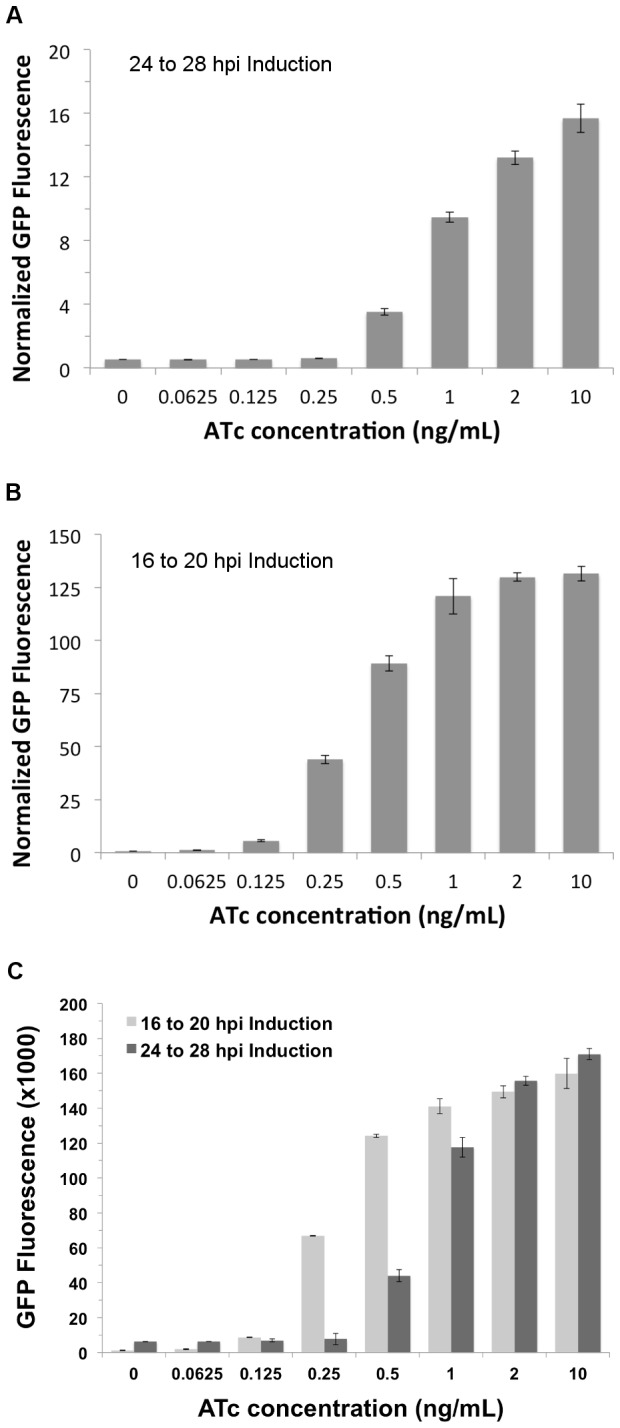
Induction of GFP with lower ATc concentrations. L929 cells were infected with pASK-GFP/mKate2-L2 transformed *C*. *trachomatis*. At 24 hpi, samples were induced for 4 hours with the indicated concentrations of ATc. After induction, soluble GFP and mKate2 in each sample was quantified using a microplate fluorometer. To normalize samples, GFP expression was divided by mKate2 expression and the resulting ratio was graphed (A). Increases observed in GFP fluorescence of samples induced with 0.25 ng/mL or higher were statistically significant compared to the 0 ng/mL sample (P < 0.01; Student’s *t* test). Induction from 16 to 20 hpi was also tested and the resulting GFP and mKate2 expression quantified as described above (B). All ATc concentrations tested for this induction period resulted in statistically significant increases in GFP expression. GFP fluorescence from the experiments shown in panels A and B are also graphed with mKate2 normalization (C). Error bars indicate standard deviation of three separate samples.

This initial set of experiments indicated little difference in rate or range of induction between 2-50 ng/ml of ATc (Figure 3). To better understand the dynamic range of ATc induction, GFP was measured after 4 hours of exposure (24 to 28 hpi) with lower concentrations of ATc using a more sensitive method of GFP detection and quantification. Instead of using fluorescence intensity from images, infected cells were lysed and fluorescence from soluble GFP and mKate2 was measured using different fluorescent filters in a microplate fluorometer. The use of *C. trachomatis* transformed with pASK-GFP/mKate2-L2 allowed the use of mKate2 expression for sample normalization (account for cell number and infection level differences) since it is under the control of a constitutively active promoter. The lowest concentration of ATc that showed a statistically significant increase in normalized GFP expression over the uninduced sample was 0.25 ng/mL, although the increase was only about 17% (Figure 5A). Much larger increases of inducible GFP were seen beginning at 0.5 ng/ml with a 1.4-2.7 step-wise increase in GFP fluorescence up to 2 ng/mL ATc, indicating more tunable control of gene expression within that range of ATc concentration. Between 2 and 10 ng/mL the increase was minimal (18% increase) suggesting that a threshold is being reached in this range. This observation is similar to the data in Figure 3 (2 *vs.* 10 ng/mL). The dynamic range of normalized GFP levels was approximately 30-fold (uninduced vs 10 ng/mL ATc). Another important observation from these experiments was the GFP levels measured in uninduced samples relative to uninfected. The GFP levels were approximately seventeen times higher than the background levels detected in the uninfected cell lysates suggesting that even in the absence of inducer, repression is incomplete and GFP accrues during the developmental cycle.

Since *C. trachomatis* has a biphasic developmental cycle in which RBs begin to asynchronously transition to EBs from 18 to 24 hpi, and given the uneven expression of GFP observed above when inducing at 24 hpi (Figure 4), it was thought that a higher dynamic range and finer control of expression might be obtained earlier in the developmental cycle when the population would be more uniform with metabolically active RBs. To test this, L929 cells infected with pASK-GFP/mKate2-L2 transformed *C. trachomatis* at 16 hpi were similarly induced (0.0625 ng/mL to 10 ng/mL) for 4 hours. In contrast to the 24-28 time points, there was very little GFP fluorescence detected in the absence of induction with approximately 2.5 times more than the uninfected background levels (not shown). Impressively, there was an increase in fluorescence for all induced samples compared to the uninduced sample, including the sample induced with 0.0625ng/mL ATc (Figure 5B). The fluorescence, as seen in the induction at 24 hpi, increased in a stepwise fashion with an almost equal doubling between 0.25-0.5 ng/mL. It does appear that after 1 ng/ml, a threshold of induction is being reached, which occurs with half as much ATc as seen with later time analyses (Figure 5A). This indicates a similar tunable control as 24-28 hpi of gene expression albeit with different range ATc concentrations (0.125-1 ng/mL in 16-20 vs 0.25 -2 ng/mL in 24-28). Also, the dynamic range at this point in the developmental cycle was higher, with an approximate 170-fold increase from 0 ng/mL to 10 ng/mL, which is much greater than the 30-fold range observed after induction at 24 hpi.

Other important observations from the studies of the two time points were the independent levels of GFP and mKate2. The infection levels for each of the time points were very similar with approximately 80-90% of the cells infected and the same concentration and stock of *Chlamydia* infectious units used. Interestingly, the levels of mKate2 during 16-20 hpi were approximately one-tenth the levels measured in 24-28 hpi (data not shown). This may be somewhat expected given the calculated 2.5-4.5 hr doubling rate of *Chlamydia* [27-29] and accumulation of consistently expressed stable mKate2. This is important to highlight given the very different normalized GFP levels observed in Figure 5A and 5B (y-axes). For comparison, un-normalized GFP fluorescence for the two time points and various induction levels were charted (Figure 5C). This shows that peak levels of induced GFP are comparable between time points, as evident in samples induced with 1, 2, and 10 ng/mL of ATc (Figure 5C). However, the inducible GFP levels for lower concentrations of ATc (0.25 and 0.5 ng/mL) during the earlier time point are considerably higher than those same induction amounts during the later time points. A feasible explanation for this difference may be due to titration of ATc in the later time points. More precisely, there is insufficient concentration of ATc molecules in these lower concentrations to relieve repression of active promoters due to ATc bound to TetR in EBs (or cells in other metabolic states for which the *tet* promoter is not active) that did not result in GFP expression. This is in contrast to the 16-20 hpi induction samples that showed that nearly all of the organisms in an inclusion were capable of responding to ATc (Figure 4B).

### GFP expression is detected relatively early in the *C. trachomatis* developmental cycle

**Figure 6:**
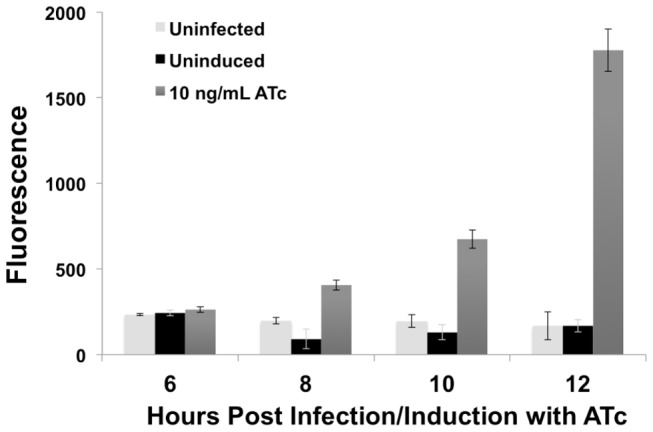
Detection of GFP induction early in the developmental cycle. L929 cell cultures infected with transformed *C*. *trachomatis* were induced at the start of infection (0 hpi). Samples were taken and lysates prepared at the indicated time points after the start of infection. GFP fluorescence was measured using a microplate fluorometer with the results quantified in arbitrary units. Error bars indicate standard deviation from two separate experiments.

After gaining access to the intracellular environment and establishing the inclusion, EBs will begin converting into RBs. Complete conversion into RBs, as evident by transcription of the majority of the genes, occurs between 6-8 hours and subsequently, RBs will begin replicating. To determine how soon inducible gene expression could be detected, GFP fluorescence was assayed between 4 to 12 hpi. Inducer was added upon infection and it was not until 8 hpi that specific GFP expression could be detected (Figure 6). Expression levels increased substantially over the next four hours with a doubling between 8-10 and almost a tripling by 12 hpi. Throughout these time points, uninduced GFP levels were minimal indicating tight repression.

### Gene expression is repressed quickly following removal of ATc

**Figure 7:**
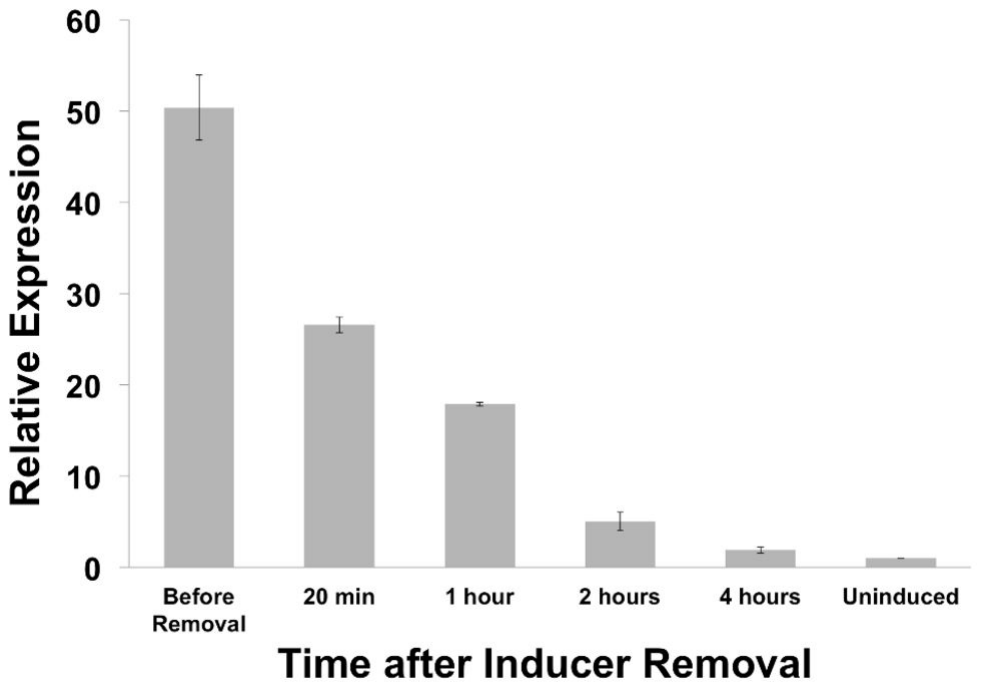
Detection of GFP gene transcription by qPCR. L929 cell cultures infected with pASK-GFP-L2 transformed *C*. *trachomatis* were induced with 10 ng/mL ATc from 20-24 hpi. The culture medium was then replaced with ATc-free medium and samples were collected at the indicated time points for processing and analysis of GFP transcription by qPCR. Relative expression levels are in comparison to the uninduced sample. Error bars indicate standard deviation from two separate experiments.

In addition to characterizing the parameters associated with gene expression induction, it is critical to determine how rapid gene expression in repressed following removal of inducer. To characterize gene repression dynamics with this system in *Chlamydia*, a relatively high inducer concentration (10 ng/mL final concentration) was added between 20-24 hpi before media was replaced without inducer was exchanged. As protein stability following repression can be extensive and will vary, GFP transcript levels were analyzed using qRT-PCR to detect immediate repression. Twenty minutes after removal of inducer, transcript levels were reduced by almost half and within 2 hours the levels were about 10% of the induced samples (Figure 7). These data support that repression occurs fairly quickly, but complete repression after maximal induction takes about 4 hours.

## Discussion

The results of this study demonstrate that the Tet control system, in combination with ATc, can be used in *C. trachomatis* for conditional gene expression. This is a major advance for the field as it will now enable numerous studies where timed and/or dose expression is critical for studying the basic biology of *Chlamydia*, host interactions, and pathogenesis. Given the many critical stages in the chlamydial developmental cycle, it is expected that inducing expression of a candidate gene at a specific point (e.g. before RB to EB conversion) will allow for more precise characterization of biological roles and functions. Moreover, incorrectly timed and dosed gene expression may have deleterious effects on the growth or development of *Chlamydia*, and it may not be even possible to obtain transformants with plasmids that constitutively express certain genes. Importantly, while gene expression from the *tet* promoter in the absence of ATc (repressed by TetR) was minimal in *Chlamydia*, leaky expression was detected after 24 hpi (Figure 5C). Although it remains to be seen whether this leaky expression will lead to challenges, the promoter can be further manipulated to decrease promoter strength and/or add additional *tetO* elements to obtain even better controlled expression.

One of the major concerns with employing the Tet system was the potential negative effect of the inducer, ATc. While ATc has not been shown to inhibit protein synthesis as tetracycline does, adverse effects on various bacterial species at higher concentrations are predicted to occur through compromised membrane integrity [17]. Most of the studies in this report used 10 ng/mL, largely due to the uncertainty of the stability of ATc in cell culture, especially during longer-term studies. At this concentration, no growth effects – progeny or inclusion size – were evident (Figure 2). However, less apparent negative effects that occur following exposure at this concentration cannot be excluded and these may be revealed as future transcriptomic and proteomic studies are performed in the presence of ATc. Also decreasing concern for potential negative effect of ATc was the effective gene induction at much lower ATc concentrations. During the early time points (16-20 hpi), GFP expression was still strong when concentrations as low as 0.25 ng/mL were applied (Figure 5C). The array of ATc concentration (0.0625-10 ng/mL) also provides an effective dynamic range for gene expression and the ability to tune expression of a gene of interest. At this time of the developmental cycle, the GFP fluorescence range was close to 130 fold (uninduced through 10 ng/mL). By comparison, regulation ranges for the Tet system vary among bacterial species with up to a 62-fold range reported in *Mycobacterium* to a 5000-fold regulation range that has been observed in *E. coli* [9,11].

One of the interesting observations in this study was the uneven expression of GFP among *C. trachomatis* cells in an inclusion when ATc was added at 24 hpi. By comparison, GFP expression was more uniform within an inclusion when ATc was added at 16 hpi. The uneven expression after 24 hpi is expected to reflect the metabolic diversity of organisms during this stage of development. Specifically, that many of the RBs are transitioning into EBs for which transcription from the *tet* promoter is either weak or not active even when TetR repression is relieved. Additionally, it may reflect an inability of ATc to effectively traverse chlamydial membranes that are undergoing reorganization during conversion and relieve transcription repression. Whatever the cause, the lack of uniformity in induction will likely need to be accounted for and may have implications for biological studies performed during this stage of the developmental cycle.

These studies also demonstrate the flexibility of the native plasmid and regulatory components. The GFP gene was placed under Tet control in an *E. coli* plasmid (pASK-IBA33) and ligated to the *C. trachomatis* L2 plasmid. pASK-IBA33 with GFP is about 4,000 bp making the completed pASK-GFP-L2 plasmid close to 11,500 bp. When mKate2 is then added, the plasmid (pASK-GFP-L2/mKate2) is around 12,200 bp – not quite twice the size of the native plasmid (~7,500 bp). Moreover, a site (between converging *pgp7* and *pgp8* genes) not previously targeted on the native *Chlamydia* plasmid was used to clone the Tet vector (pASK-IBA33). We did not assess the plasmid copy number between native *Chlamydia* plasmid and the modified vector; however, maintenance of this plasmid was stable for many culture passes even after removal of selection (data not shown). The Tet repressor gene (*tetR*) or the *tetA* promoter was not modified (e.g. codon or promoter optimized) for the Tet control system to be functional in *C. trachomatis*. Additionally, the *tetR* gene is encoded downstream within an operon with *bla* (β-lactamase). The promoter driving this operon was clearly functional as TetR was expressed and repressed transcription. Additionally, the concentration of ampicillin used for selection were not reduced relative to our selection levels for the original pGFP::SW2 plasmid, supporting strong expression of β-lactamase from pASK-GFP-L2/mKate2. As the minimal genetic components for maintenance and replication of the *Chlamydia* plasmid continue to be characterized [19,30], the extent of amount and content of foreign DNA that can be added will likely expand as will our ability to utilize the vector for genetic studies.

Another observation of note is the functionality of mKate2 in *Chlamydia*. mKate2 was selected due to the spectral separation from GFP and other fluorescent proteins (far-red fluorescence), low associated background fluorescence, and overall brightness [31]. The mKate2 gene was codon optimized for *Chlamydia* and a chlamydial promoter (*dnaK*) was employed to generate strong constitutive expression. As indicated in the Materials and Methods, expression of mKate2 using the wild-type *dnaK* core promoter was apparently toxic to *Chlamydia* as growth was affected. The wild-type *dnaK* promoter is almost a canonical σ^70^ promoter and was weakened using the observations of Schaumburg et al [32] which allowed for mKate2 expression and viable and infectious *Chlamydia* that are easy to detect by fluorescence microscopy. Agaisse and Derre demonstrated that mCherry and CFP (cyan) are also functional in *Chlamydia* [5]. In combination, these expand the fluorescent protein repertoire for *Chlamydia* and will enable numerous comparative sub-cellular localization and expression analyses.

In summary, these data support that a system for conditional gene expression has been adapted to and is functional in *Chlamydia*. While conditional gene expression is an essential molecular tool, the ability to disrupt gene expression through deletion or repression is also important. The Tet system has the future potential flexibility for the use of the revTetR repressor that allows for inducible repression at sites engineered with *tetO* sequences [18]. Alternatively, the Tet system described herein could be used for inducing expression of interfering molecular components such as anti-sense RNAs, dominant negative protein, and CRISPR associated mechanisms (i.e. Cas9), and sequence specific TALEN proteins. Furthermore, the conditional gene expression system described will also be a highly valuable tool for genetic complementation studies. In all, the continued transilience of genetics in *Chlamydia*, including conditional gene expression, holds much promise to better understand the basic biology and pathogenesis of *Chlamydia*.

## Materials and Methods

### Ethics Statement

All genetic manipulations, containment parameters, and biological experiments involving *Chlamydia* and *E. coli* were approved by the University of Kansas rDNA and Biologic Use Committees and are in compliance with NIH guidelines for research involving recombinant DNA molecules.

### Shuttle vector construction

The GFP gene from pGFP::SW2, which was shown to be functional in *C. trachomatis* [4] was amplified by PCR using the Phusion High Fidelity Polymerase kit (Thermo Scientific) and inserted into pASK-IBA33plus (IBA, Göttingen) at the *EcoR*I and *Pst*I sites (see Figure S1 for primer and final construct sequences) using the In-Fusion HD cloning kit from Clontech. The CAT gene fused to GFP in pGFP::SW2 was not included in pASK-GFP, however, the translation start and stop codons from pASK were used for GFP, so the resulting gene product contained additional amino acids at the N-terminus and C-terminus, including a C-terminal His6 tag. The entire pASK-GFP plasmid was amplified by PCR with primers that encoded a *Kas*I site at one end and an *Apa*I site at the other end. The wild-type plasmid of *C. trachomatis* L2 was similarly amplified by PCR and both PCR products were digested with *Kas*I and *Apa*I, then ligated to generate the shuttle vector pASK-GFP-L2. The f1 origin of pASK was disrupted, but we attempted to avoid any disruption of the open reading frames or promoters on the L2 plasmid by creating the junction between the convergent genes *pgp7* and *pgp8* (see Figure 1). Construction of pASK-GFP/mKate2-L2 also started with pASK-IBA33, but in this case, the multiple cloning site of pASK was replaced with a single *Age*I site such that any insert would provide the start codon immediately downstream of the *Age*I junction. The GFP gene was then inserted at this *Age*I site placing it under control of the *tetA* promoter, and the resulting GFP product would have no vector-encoded amino acids at either terminus. Also, the *Age*I site was recreated at the 5’ end of GFP, but not at the 3’ end where it was replaced with an *Eag*I site. The resulting plasmid was ligated to the wild-type plasmid of *C. trachomatis* L2, as described above. Additionally, the gene encoding the fluorescent protein mKate2 (codon optimized for *C. trachomatis*) under control of a weakened *C. trachomatis dnaK* (more specifically, the promoter of the *hrcA-grpE-dnaK* operon) core promoter was inserted at the *Kas*I site (see Figure S1 for sequences). Initially, mKate2 expression under control of a wild-type *dnaK* core promoter was tested, but this level of expression appeared to have negative effects on cell growth, so a substitution was made to weaken the *dnaK* promoter, based on information from Schaumburg and Tan [32]. The *Kas*I site was recreated at the 3’ end of mKate2, but not at the 5’ end. Oligonucleotides were synthesized by Integrated DNA Technologies (Coralville, IA).

### Cell Culture and *C. trachomatis* propagation

Murine L929 fibroblast cells (ATCC CCL-1) were used in this study. Cells were grown at 37°C under 5% CO_2_ humidified conditions in RPMI 1640 media supplemented with 0.3 mg/mL L-glutamine, 5% (vol/vol) fetal bovine serum (FBS) and 10 µg/mL gentamycin. *C. trachomatis* serovar L2 (LGV 434) was propagated in L929 cells grown in suspension culture and purified at 48 hpi using the following method. Infected host cells were collected by centrifugation at 1380 x g for 10 minutes at 4°C, washed once with HBSS, then resuspended in HBSS for sonication on ice with the Fisher Sonic Dismembrator 100 in 15 second sets (1 minute between sets) for a total of 90 seconds of sonication. The sonicated suspension was centrifuged at 150 x g for 10 minutes at 4°C and the resulting supernatant was transferred to fresh tubes for centrifugation at 28,000 x g for 30 minutes at 4°C. The resulting pellet was resuspended in SPG buffer (219 mM Sucrose, 3.7 mM KH_2_PO_4_, 8.5 mM Na_2_HPO_4_, and 4.9 mM L-glutamate; pH 7.4) and aliquots of the seed prep were stored at -80°C.

Transformation of the *C. trachomatis* seed prep was performed as described by Wang, et al [4] with the following differences. The amount of *C. trachomatis* seed prep used in the transformation reactions was determined empirically to obtain a high level of infection without excessive cytotoxicity. L929 cells were used as the host cells and ampicillin was used for selection. Ampicillin was used at a concentration of 1 μg/mL in the initial round of selection, but increased to 10 μg/mL after subsequent passage.

Infection of L929 cells with wild-type or shuttle vector-transformed *C. trachomatis* L2 was carried out as follows. L929 cells were seeded one to two days in advance of infection and grown to 50-100% confluency. *C. trachomatis* seed preps, stored in SPG buffer at -80°C, were thawed and diluted in HBSS just before infection. The cell culture medium was aspirated from L929 cell cultures and the diluted *C. trachomatis* cell suspensions were added to appropriate samples and incubated at room temperature for 2 hours. The amount of *C. trachomatis* seed prep used in the infections was determined empirically to obtain 50-75% infection levels. After two hours of incubation at room temperature, the *C. trachomatis* suspension in HBSS was aspirated and cell culture medium containing ampicillin was added. The cultures were then placed at 37°C under 5% CO_2_, which was considered 0 hpi.

### Testing of anhydrotetracycline sensitivity

Infected L929 cells cultured in 8-well µ-Slide plates were treated with ATc concentrations between 0.1 and 1.0 µg/mL from the start of the infection through fixation. At 24 hpi, cells were fixed with methanol and stained with the *Chlamydia trachomatis* Culture Confirmation Test kit (Trinity Biotech, Wicklow, Ireland), which utilizes a FITC-conjugated anti-MOMP primary antibody and Evans Blue host cell albumin stain. The cells were additionally stained with DAPI (1 µg/mL) for 15 min. Wells were washed with PBS, then 90% glycerol (in 0.1 M Tris, pH 9) was added to minimize photobleaching during imaging. Imaging was performed using an Olympus IX71 inverted fluorescence microscope (Center Valley, PA) with a 40X objective with a numerical aperture of 0.55. Infected cell counts were determined using Cell Profiler and Cell Profiler Analyst, based on the pipeline described in Osaka, et al [24] with minor modifications to additionally determine inclusion size. To assess the effect of ATc on formation of infectious progeny, infected L929 cells cultured in a 24-well plate were treated with ATc concentrations between 10 and 100 ng/mL. At 24 hpi, the host cells were lysed by hypotonic rupture to collect infectious progeny. For hypotonic lysis, culture medium was aspirated and cells were washed for 5 seconds with sterile water, then 1 mL fresh sterile water was added for 1 min followed by the addition of 250 µl of 5x SPG buffer to stop hypotonic lysis. Wells were then scraped using a pipet tip and the lysate was transferred to a tube containing sterile glass beads (1.0 mm in diameter) and vortexed for 45 sec for further lysis. Cell lysates were then serially diluted two-fold and used to infect a fresh monolayer of L929 cells in a 96-well tissue culture plate in the absence of ATc. The infected cultures were fixed at 24 hpi and stained as described above for enumeration.

### Live imaging

L929 cells in 8-well µ-Slide plates were infected with transformed *C. trachomatis* as described above. At indicated times, ATc was added to the appropriate wells. Images were captured by epifluorescense after induction. Imaging was performed using SlideBook (Intelligent Imaging Innovations, Inc., Denver, CO) software and an Olympus IX81 inverted fluorescence microscope (Center Valley, PA) with a Hamamatsu EM-CCD camera. Images of cultures infected with pASK-GFP-L2 transformed *C. trachomatis* were taken with a 40X objective with a numerical aperture of 0.95. Images of cultures infected with pASK-GFP/mKate2-L2 transformed *C. trachomatis* were taken with a 100X objective with a numerical aperture of 1.45. For quantification of GFP expression from microscopy images, the Otsu segmentation algorithm within the Slidebook software was used.

### GFP quantification by microplate fluorometer

L929 cells in 6 well plates were infected with pASK-GFP-L2 transformed *C. trachomatis*, as described above. For samples tested at 6, 8, 10, and 12 hours post infection, ATc at a final concentration of 10 ng/mL was added to the cell media at 0 hpi. The cells were collected at the indicated time points by trypsinization followed by the addition of Dulbecco’s Phosphate Buffered Saline (DPBS, Mediatech Inc., Manassas, VA). The cell suspension was centrifuged at 1380 x g for 10 minutes at room temperature. The resulting pellet was resuspended in 1 mL cold RIPA buffer (10 mM Tris-HCl pH 8.0, 1 mM EDTA, 1% (v/v) Triton X-100, 0.1% (w/v) SDS, 0.1% (w/v) sodium deoxycholate, 140 mM NaCl, 5 mM DTT) and incubated on ice for 30 minutes. The lysate was then centrifuged at 15,000 x g for 20 minutes at 4°C. The supernatant from the final centrifugation was collected and plated in quadruplicate in a 96 well, solid bottom, black-walled plate (Costar, Corning Incorporated, Corning, NY). GFP was quantified using a BioTek Synergy 2 Multi-Mode Microplate reader with Gen5 1.11 software and the optics position set at “Top 50%.” A 485/20, 528/20 filter was used for reading fluorescence emission of GFP. A Bradford assay was performed using a portion of the final supernatant to determine the total protein concentration of each sample, which was 1.2 mg/mL +/- 10%. The procedure was altered for the experiments with pASK-GFP/mKate2-L2 transformed *C. trachomatis*. Infections were set up in 12 well plates and at the appropriate times, the culture medium was aspirated and 0.5 mL cold RIPA buffer was added to the infected monolayer. The plate was incubated at 4°C for 30 minutes, then the lysate was collected and centrifuged at 15,000 x g for 20 minutes at 4°C. The supernatant was then transferred to the 96 well plate for reading. Quantification of GFP was performed as described above, and expression of mKate2 was measured using a 540/25, 620/40 filter on the BioTek Synergy 2 Multi-Mode Microplate reader. The Synergy 2 microplate reader has >6 decades of dynamic range for fluorescence intensity.

### Quantitative PCR

Infected L929 cell cultures in 6 well plates were induced with 10 ng/mL ATc from 20 to 24 hpi, then washed once before addition of pre-warmed culture medium without ATc. The samples were then returned to the incubator until collection at the indicated times. Collection of samples, homogenization with TRIzol (Invitrogen), and phase separation for RNA isolation were carried out as described in the TRIzol reagent protocol. However, after addition of isopropanol to precipitate the RNA, the suspension was added to an RNeasy (Qiagen) Miniprep column rather than pelleting the RNA precipitate by centrifugation. The RNeasy procedure was carried out as directed in the kit protocol. The final eluate was treated with DNase using the TURBO DNA-free DNase Treatment kit (Invitrogen) and the resulting sample was used to generate cDNA using the High Capacity RNA-to-cDNA Kit (Applied Biosystems). Quantitative PCR was set up with SYBR Green PCR Master Mix (Applied Biosystems) and gene specific primers that were designed using Primer3 web-based software [33]. Data collection and analysis were done using the StepOne software and StepOnePlus Real-Time PCR system (Invitrogen). Samples were normalized based on *gyrB* and *secY* transcription levels.

## Supporting Information

Video S1 **L929 cell cultures infected with pASK-GFP-L2 transformed *C. trachomatis* were induced with 10 ng/mL **ATc** **at** 24 hpi.** Image capture was started 15 minutes after addition of ATc and images were captured every 30 minutes for 3 hours and combined to make a video.
(MP4)

Video S2 **L929 cell cultures infected with pASK-GFP/mKate2-L2 transformed *C. trachomatis* were induced with 10 ng/mL **ATc** **at** 24 hpi.** Image capture was started 15 minutes after addition of ATc and images were captured every 15 minutes for 6 hours and combined to make a video.
(MP4)

Video S3 **L929 cell cultures infected with pASK-GFP/mKate2-L2 transformed *C. trachomatis* were induced with 10 ng/mL **ATc** **at** 16 hpi.** Image capture was started one hour after addition of ATc and images were then captured every 30 minutes for 5 hours and combined to make a video.
(MP4)

Figure S1 **Primer sequences used to construct shuttle vectors are shown.** The final sequences of the two shuttle vectors used in this study are also shown, although only inserted genes and the adjacent vector sequences were verified by sequencing.
(DOCX)
